# The LIM-Only Protein FHL2 Reduces Vascular Lesion Formation Involving Inhibition of Proliferation and Migration of Smooth Muscle Cells

**DOI:** 10.1371/journal.pone.0094931

**Published:** 2014-04-15

**Authors:** Kondababu Kurakula, Mariska Vos, Iker Otermin Rubio, Goran Marinković, Reinhard Buettner, Lukas C. Heukamp, Jan Stap, Vivian de Waard, Claudia M. van Tiel, Carlie J.M. de Vries

**Affiliations:** 1 Department of Medical Biochemistry, Academic Medical Center, University of Amsterdam, Amsterdam, The Netherlands; 2 Institute of Pathology, University of Cologne, Cologne, Germany; 3 Department of Cell Biology and Histology, Academic Medical Center, University of Amsterdam, Amsterdam, The Netherlands; The University of Tennessee Health Science Center, United States of America

## Abstract

The LIM-only protein FHL2, also known as DRAL or SLIM3, has a function in fine-tuning multiple physiological processes. FHL2 is expressed in the vessel wall in smooth muscle cells (SMCs) and endothelial cells and conflicting data have been reported on the regulatory function of FHL2 in SMC phenotype transition. At present the function of FHL2 in SMCs in vascular injury is unknown. Therefore, we studied the role of FHL2 in SMC-rich lesion formation. In response to carotid artery ligation FHL2-deficient (FHL2-KO) mice showed accelerated lesion formation with enhanced Ki67 expression compared with wild-type (WT)-mice. Consistent with these findings, cultured SMCs from FHL2-KO mice showed increased proliferation through enhanced phosphorylation of extracellular-regulated kinase-1/2 (ERK1/2) and induction of CyclinD1 expression. Overexpression of FHL2 in SMCs inhibited CyclinD1 expression and CyclinD1-knockdown blocked the enhanced proliferation of FHL2-KO SMCs. We also observed increased CyclinD1 promoter activity in FHL2-KO SMCs, which was reduced upon ERK1/2 inhibition. Furthermore, FHL2-KO SMCs showed enhanced migration compared with WT SMCs. In conclusion, FHL2 deficiency in mice results in exacerbated SMC-rich lesion formation involving increased proliferation and migration of SMCs via enhanced activation of the ERK1/2-CyclinD1 signaling pathway.

## Introduction

Vascular smooth muscle cells (SMCs) provide the vessel wall structural integrity and the capacity to modulate blood supply through vasodilatation and vasoconstriction. Arteries comprise multiple layers of SMCs that are organized in the so called media of the vessel wall, which is at the luminal side of covered by a single layer of endothelial cells. In vascular diseases such as atherosclerosis and (in-stent) restenosis after percutaneous coronary interventions, smooth muscle cells (SMCs) play a crucial role [1]. SMCs undergo a phenotypic switch upon activation and are often referred to as ‘synthetic or activated SMCs’ in contrast to ‘normal’, quiescent SMCs that exhibit the contractile phenotype [1], [2]. Synthetic SMCs show enhanced proliferation and migration and are pro-inflammatory. Furthermore, these activated SMCs synthesize excessive amounts of extracellular matrix, whereas expression of SMC-specific marker genes is reduced [1], [3]–[5]. SMC proliferation and migration is known to contribute to the development of vascular restenosis [3], [6]–[8].

The LIM-only protein FHL2/DRAL/SLIM3 (Four and Half LIM domain protein 2) is a member of the FHL protein family. FHL2 is known to interact with a number of proteins, and functions as a crucial coactivator or corepressor of the interacting partners. The strength and activating or antagonizing function of FHL2 strongly depends on the cell-type and cellular context in which FHL2 interacts with other proteins [9], [10]. FHL2 modulates the activity of transcription factors, such as the androgen receptor (AR), NF-κB, cAMP-responsive element binding protein (CREB) and Nur77, in a range of physiological and pathological processes, among which proliferation, migration, differentiation and apoptosis [9], [11]–[13]. FHL2 is highly expressed in heart and skeletal muscle as well as in vascular cells, including SMCs, but also at low levels of expression in other cell types and tissues [9], [11], [12], [14]. Although FHL2-deficient mice maintain normal cardiac function they display cardiac hypertrophy in response to β-adrenergic stimulation [15]. FHL2 has been described to prevent extracellular signal-regulated kinase (ERK)-induced cardiac hypertrophy through binding and inhibiting ERK in cardiomyocytes [13]. As a serum-response factor (SRF) target gene, FHL2 antagonizes RhoA and bone morphogenetic protein (BMP) signaling pathway-mediated induction of SMC differentiation markers such as smooth muscle α-actin (SM α-actin), calponin and SM22-α [10], [12]. In contrast with these data, FHL2 has been described to enhance protein stability of myocardin-like proteins resulting in enhanced SMC marker genes [16]. Deletion of FHL2 has been associated with resistance to atherogenesis, possibly via regulation of its function in endothelial cells [17]. Restenosis and in-stent restenosis after angioplastic intervention in man is characterized by excessive SMC proliferation and may be considered a typical SMC pathology.

The function of FHL2 in vascular repair during restenosis has not been studied and based on the reported functions of FHL2 in SMC gene expression in cultured cells the outcome of *in vivo* injury studies was unpredictable. In the current study, we show to best of our knowledge for the first time that FHL2 deficiency in mice results in enhanced SMC-rich lesion formation following vascular injury by carotid artery ligation. The underlying cause involves increased proliferation and migration of vascular SMCs deficient for FHL2, showing enhanced ERK1/2 activation and CyclinD1 expression. Based on our data, we propose that the intricate regulatory function of FHL2 in the complex phenotypic changes of SMCs upon vascular injury contributes to inhibition of vascular lesion formation.

## Materials and Methods

### Animals and Ethics statement

All experiments were approved by an independent animal ethic committee of the Amsterdam Medical Center, University of Amsterdam, The Netherlands (permit number DBC102226) and were carried out in compliance with guidelines issued by the Dutch government. All animals were cared for and maintained under the strict supervision and guidelines of the animal ethic committee. All surgery was performed under midazolam and medetomidine hydrochloride anesthesia, and all animals were sacrificed under ketamine/xylazine anaesthesia. Every effort was made to minimize both the suffering of the animals and the number of animals utilized. FHL2-deficient mice were generated by R. Bassel-Duby (University of Texas Southwestern Medical Center, Dallas, TX). FHL2-KO mice were bred onto a C57BL/6 background for >11 generations.

### Left carotid artery ligation

Mouse carotid artery ligation was performed as previously described [18]. Briefly, the left common carotid artery of 12–13 weeks old male wild-type and FHL2-KO mice was ligated just proximal to the carotid bifurcation. Before surgery, mice were anaesthetized with an intra-peritoneal injection of a mixed solution of 5 mg/kg midazolam (Dormicum, Roche), 0.5 mg/kg medetomidine hydrochloride (Domitor, Pfizer) and 0.05 mg/kg fentanyl (Bipharma). The right carotid artery served as an uninjured contralateral control. Mice were sacrificed at 0, 1, 2, and 4 weeks after carotid ligation (n = 7 for 1 and 2 weeks and n = 14 for 4 weeks). Prior to sacrifice, the body weight of all mice was measured followed by anaesthesia by intra-peritoneal injection with 80 mg/kg ketamine (Nimatek; Eurovet) and 5 mg/kg xylazine (Sedamun, Eurovet) and perfused with saline. The left and right carotid arteries were placed in Trizol (Invitrogen) for RNA isolation or embedded in paraffin for histological analysis. Morphometric analysis was performed on at least 5 sections per mouse at a fixed position proximal from the ligation site; 1 week at 1.7 mm, 2 weeks ligation at 2.0 mm and 4 weeks at 2.3 mm.

### Immunohistochemistry

Paraffin sections were deparaffinized and rehydrated. Hematoxylin/Eosin (HE) and Lawson stainings for measuring neointima formation were performed as described previously [19]. Proliferating cells were detected with an antibody against Ki67, antigen retrieval was performed at pH 6.0 and sections were blocked with Ultra-V-block (Thermo Scientific). The first antibody was incubated overnight at 4°C followed by an HRP-conjugated secondary goat anti-rabbit antibody. DAB substrate was used for detection. After counterstaining with hematoxylin all the sections were embedded in pertex (HistoLab). Neointimal area was quantified using Leica QWin V3 software.

### Preparation of mouse aortic SMCs

Mouse aortas were harvested and aortic SMCs were prepared as described previously [20]. Briefly, aortas were harvested from 8–12 weeks old mice, perivascular fat was removed and aortas were digested in 1 g/L collagenase II (Sigma C), 0.25 g/L elastase (Sigma), 1% penicillin/streptomycin (PAA Laboratories GmbH), and 1 g/L soybean trypsin inhibitor (Sigma) in Hank's Balanced Salt Solution (Gibco) for 20 minutes at 37°C. Following digestion the adventitia was carefully removed and the intimal surface was gently scraped with fine forceps. Aortas were cut into ∼0.5 mm pieces and placed in enzyme solution again for 1 hour. Disaggregated medial SMCs were then grown and maintained in DMEM/F12 medium [Gibco] containing 20% fetal calf serum [Gibco] and 1% penicillin/streptomycin). For some experiments as described, SMCs were switched to serum-free medium (DMEM/F12, 1% penicillin/streptomycin). All *in vitro* experiments were performed in at least three different SMC isolations, which were maximally used up to passage no. 8.

### BrdU Incorporation Assay

Cultured aortic SMCs were seeded in 96-well plates at a density of 3×10^3^ cells/well and incubated overnight in full medium. The cells were made quiescent by incubation in medium without FCS for 48 h, then FCS (20% v/v) was added and incubated for another 24 h. DNA synthesis was measured by the BrdU incorporation assay (Roche) according to the manufacturer's instructions. Briefly, the cells were incubated with BrdU for 16 h, fixed, and incubated with conjugated anti-BrdU antibody, and finally colorimetric analysis was performed with an ELISA plate reader. Each experiment (in quadruplicate) was repeated at least four times. Cells were pre-treated for overnight with PD98059 (an ERK1/2 inhibitor, Sigma) at a final concentration of 25 µM.

### Western blot analysis

SMCs were lysed in RIPA buffer with protease inhibitor cocktail (Sigma). The cell lysates were separated by SDS-PAGE and transferred onto PVDF membranes (Millipore). After protein transfer, membranes were blocked with Odyssey blocking buffer (LI-COR) and incubated with the appropriate primary antibodies and fluorescently conjugated secondary antibodies, followed by scanning using the Odyssey Infrared Imaging System (Licor Biosciences). Antibodies applied in this study were alpha-tubulin (Cedarlane laboratories), anti-ERK1/2 (Santa Cruz), and anti-pERK1/2 (Santa Cruz).

### Generation of lentiviral particles and infection

Recombinant lentiviral particles encoding FHL2, shRNAs targeting CyclinD1 and shRNAs targeting FHL2 were produced, concentrated, and titrated as described previously [11]. Two different mouse shRNAs that target different regions in the CyclinD1 mRNA (shCyclinD1 #1 target sequence: CTTTCTTTCCAGAGTCATCAA and shCyclinD1 #2 target sequence: CCCTGACACCAATCTCCTCAA) and in the FHL2 mRNA (shFHL2 #1 target sequence: GATGGGAAGATGGTTTGGAAT and shFHL2 #2 target sequence: CTGTGACTTGTACGCTAAGAA) were used for generation of lentiviruses. Cultured aortic SMCs were infected with recombinant lentivirus for 24 h after which the medium was refreshed and the cells were cultured for another 24 h. After this incubation period, cells were serum starved for another 36 h before harvesting. Transduction efficiency was determined by immunofluorescence and qRT-PCR.

### RNA Extraction and quantitative RT-PCR

Total RNA was harvested from cells using the Total RNA mini kit (Bio-Rad) or Trizol reagent (Invitrogen) according to the manufacturer's instructions. cDNA was made using the iScript cDNA synthesis kit (Bio-Rad). Real-time reverse transcription PCR was performed using the MyIQ system (Bio-Rad) and the following primers: CyclinD1 forw: 5′-GGCCACTGAGGAGGAGGGGG-3′, CyclinD1 rev: 5′-TCCCCAAGGGGGACGTCGTC-3′, Ki67 forw: 5′-CAGTACTCGGAATGCAGCAA-3′, Ki67 rev: CAGTCTTCAGGGGCTCTGTC-3′, PCNA forw: 5′-AATGGGGTGAAGTTTTCTGC-3′, PCNA rev: 5′-CAGTGGAGTGGCTTTTGTGA-3′. Cells were pre-treated overnight with PD98059 at a final concentration of 25 µM. Acidic ribosomal phosphoprotein P0 was determined as an internal control for cDNA content of the samples (P0 forw: 5′-GGACCCGAGAAGACCTCCTT-3′, P0 rev: 5′-GCACATCACTCAGAATTTCAATGG-3′).

### In vitro scratch-wound assays

Cultured aortic SMCs were seeded in 6-well plates in DMEM/F12 medium containing 20% FCS. After 16 h, confluent cells were serum starved for 48 h and scratched with a 100 µl pipette tip. The cells were stimulated with 25 ng/ml of PDGF-BB (TEBU-BIO) and the medium in the wells was layered with mineral oil (Sigma M3516) to prevent evaporation. Cell migration was assayed during 48 h after scratching and stimulation with PDGF-BB. Six areas were chosen randomly for taking images every 10 min under a Leica live-cell microscope (DMIRBE) and the relative closure distance was measured. Images were captured with a digital camera (Apogee) and movies were generated. Quantification was made using custom-made software. Each experiment (in duplicate) was repeated at least three times.

### In vitro migration assay using trans-well chambers

SMC migration was evaluated in a trans-well chamber according to the method by Goncharova *et al.* with modification [21]. Briefly, confluent cells were serum starved for 48 h, washed with PBS, and then detached by trypsin. Cells were labeled fluorescently using the CellTrace CFSE Cell Proliferation Kit (Molecular Probes, Invitrogen). Cells were plated at a density of 2×10^5^ cells/well in 24-well trans-well plates (8-µm pore size; BD Falcon) in serum-free DMEM/F12 medium and serum-free medium was also added to the lower chamber. Cells were allowed to migrate for 3 h under 5% CO_2_ and 21% O_2_ in a humidified incubator at 37°C and fluorescence was measured using a cell-based fast kinetic microplate reader (NOVOstar, BMG-labtech). Each condition was repeated in triplicate.

### Luciferase reporter assays

Transient transfection and reporter assays were carried out in serum starved SMCs with the indicated reporter plasmids using Fugene6 transfection reagent (Roche) according to the manufacturer's protocol. The level of promoter activity was evaluated by measurement of firefly luciferase activity. pRL-TK Renilla reporter plasmid (Promega) was co-transfected as an internal control. Luciferase activity was measured by using the Dual Luciferase Assay System and Glomax Multi detection system as described by the manufacturer (Promega). pGL3-CyclinD1 reporter vector was a kind gift from Dr. J J Molenaar (Academic Medical Center, University of Amsterdam, Amsterdam, The Netherlands) and has been described [22]. To increase basal CyclinD1, serum-starved SMCs were stimulated with FCS. Cells were pre-treated overnight with PD98059 at a final concentration of 25 µM. A minimum of three independent transfections were performed and all assays were repeated at least three times.

### Statistical analysis

Data are reported as mean±SD unless otherwise specified. Results were analyzed with the unpaired Student's *t*-test. *P* values <0.05 were considered as statistically significant.

## Results

### Deficiency of FHL2 exacerbates vascular lesion formation in vivo

**Figure 1:**
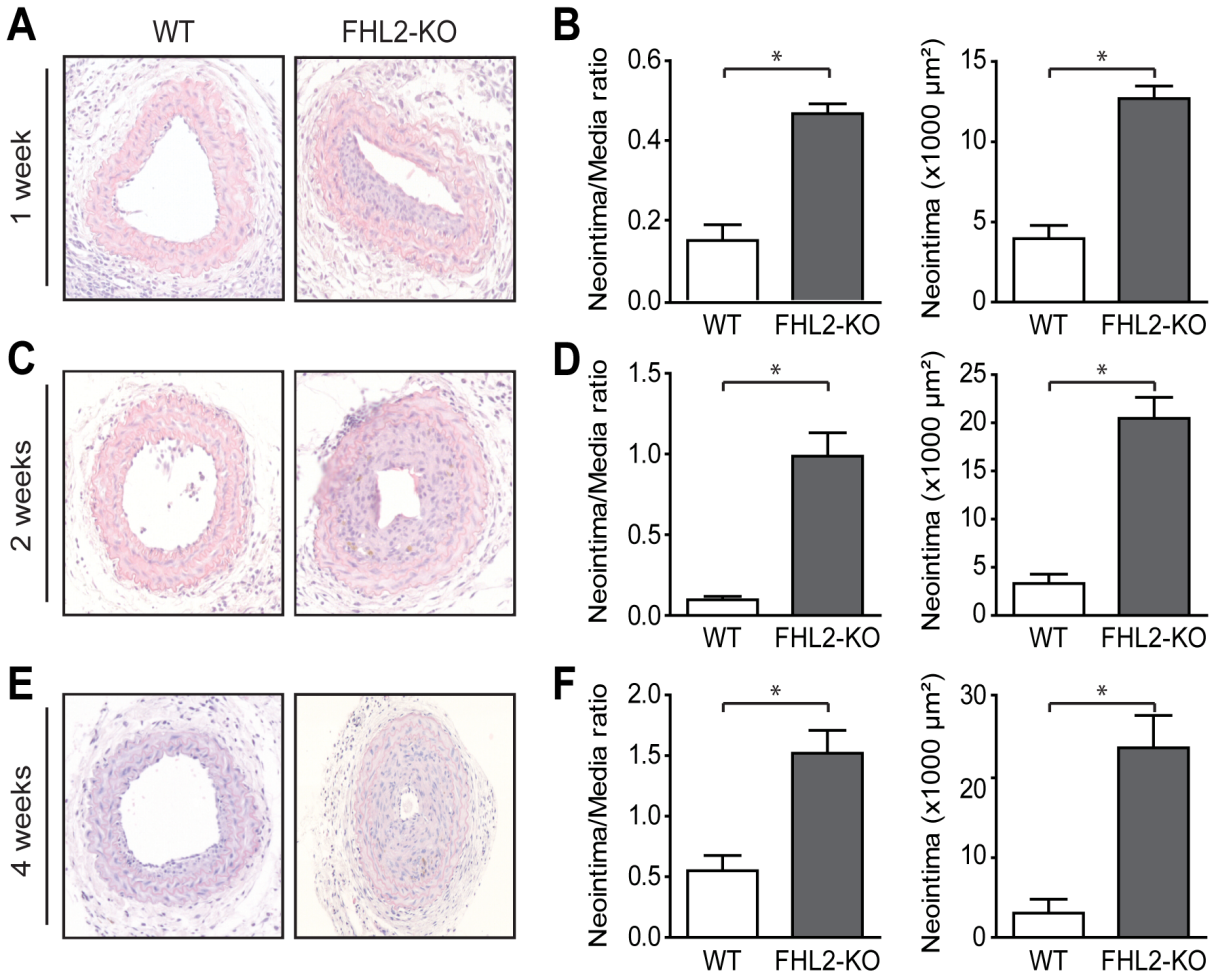
Deficiency of FHL2 accelerates neointima formation after carotid artery ligation. **A, C** and **E**; Representative cross sections of hematoxylin/eosin-stained carotid arteries from WT and FHL2-KO mice ligated for 1 (A), 2 (C) and 4 weeks (E). **B, D** and **F**; Quantitative analysis of neointima/media ratio and neointimal area in histological sections from WT and FHL2-KO mice ligated for 1 (B), 2 (D) and 4 weeks (F), revealed increased lesion formation in FHL2-KO mice. n = 7 for 1 and 2 weeks and n = 14 for 4 weeks. Three consecutive sections per mouse at each location were employed in the analysis. Lesions were characterized at 1.7, 2.0 and 2.3 mm from the reference point at 1, 2 and 4 weeks, respectively. Values are mean±SEM. *P<0.05 for FHL2-KO versus WT mice.

FHL2 has been associated with SMC proliferation and differentiation, however, in cultured cells conflicting data are available on its function in SMC phenotype modulation [9]–[12]. Therefore, we sought to determine whether FHL2 is functionally involved in SMC-rich lesion formation *in vivo* and performed carotid artery ligations in WT and FHL2-KO mice. Both at the onset of the experiment and 4 weeks after ligation the white blood cell counts were similar in WT and FHL2-KO mice (Figures S1A–B). Vascular lesions in the ligated carotid arteries were analyzed after 1, 2 and 4 weeks. Quantitative morphometry revealed significantly more lesion area (neointimal area) and an increased neointima/media ratio after 1, 2 and 4 weeks in FHL2-KO mice (Fig. 1A–F). Based on these data, we concluded that endogenous FHL2 suppresses vascular intima formation after injury of the carotid artery.

### FHL2 deficiency promotes lesion formation via enhanced cell proliferation in vivo

**Figure 2:**
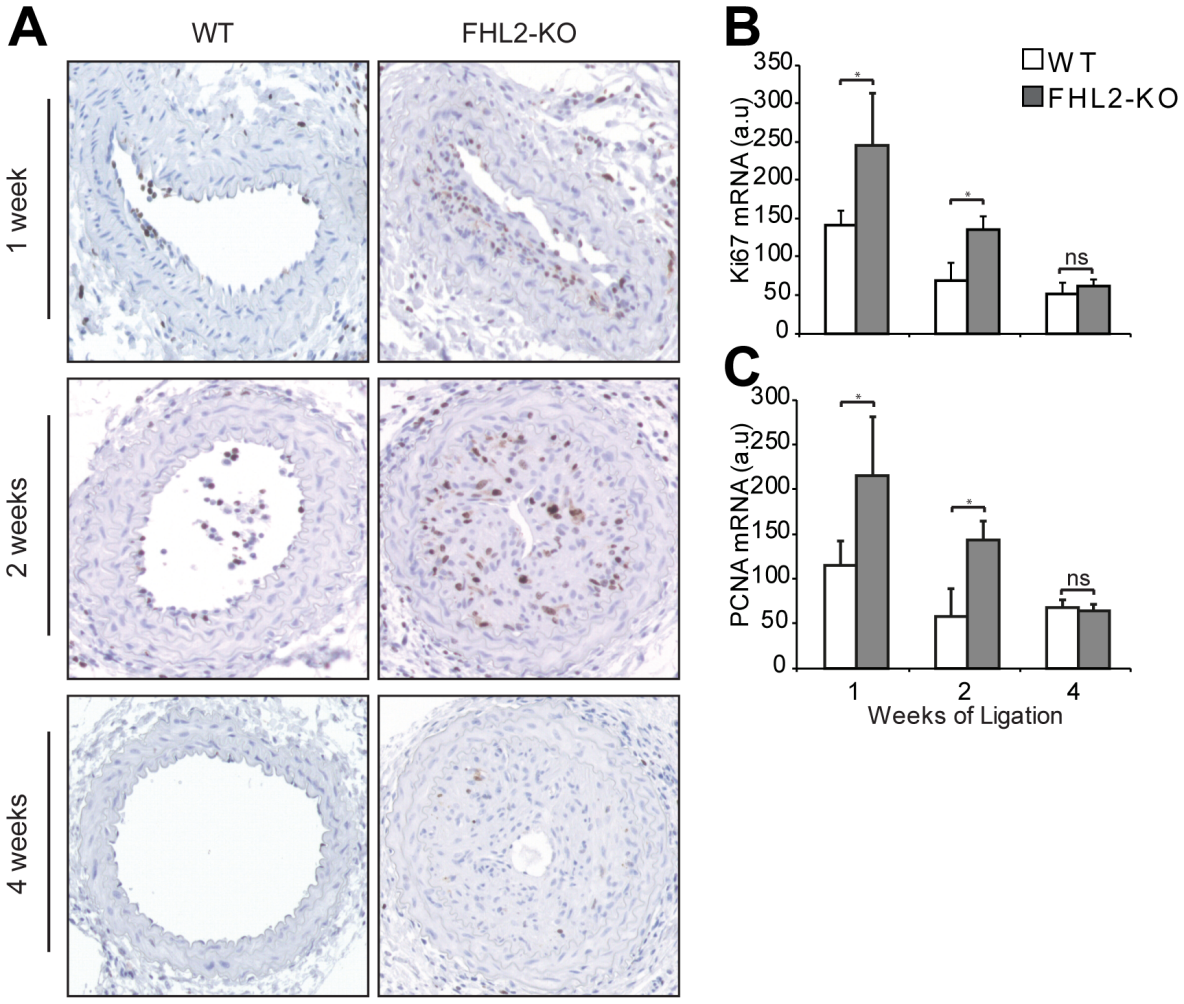
FHL2 deficiency enhances cell proliferation *in vivo*. **A**, To assess the extent of proliferation in the vascular lesions, representative sections of injured carotid arteries from WT and FHL2-KO mice ligated for 1, 2 and 4 weeks were immunostained for Ki67. n = 7 for 1 and 2 weeks and n = 14 for 4 weeks. **B–C**, qRT-PCR was performed to assess mRNA expression of Ki67 (B) and PCNA (C) in the ligated vessels from WT and FHL2-KO mice for the indicated time periods. Data are means±SD. *P<0.05 for FHL2-KO versus WT mice.

In line with the original description of this injury model immunohistochemical staining for SM α-actin revealed that SMCs were the predominant cell type in the neointimal area after carotid artery ligation regardless of the genotype, whereas only limited numbers of macrophages were present (data not shown) [18]. To test for enhanced SMC proliferation as a cause for the increased neointima formation in FHL2-KO mice, we stained sections for Ki67 at 1, 2 and 4 weeks after ligation. Most Ki67-positive cells were present after 1 and 2 weeks of ligation. Injured vessels of FHL2-KO mice contained a higher number of Ki67-positive cells compared with those of WT mice after 1 and 2 weeks of ligation (Figs. 2A). In addition, we determined mRNA expression of Ki67 and PCNA in the ligated vessels from WT and FHL2-KO mice. We found that expression of both Ki67 and PCNA mRNA was significantly higher in FHL2-KO compared to WT at 1 and 2 weeks after ligation (Figs. 2B–C). These findings support the observation that enhanced SMC proliferation in FHL2-KO mice promotes neointima formation after carotid artery ligation.

### FHL2 deficiency enhances SMC proliferation involving ERK1/2 and CyclinD1

**Figure 3:**
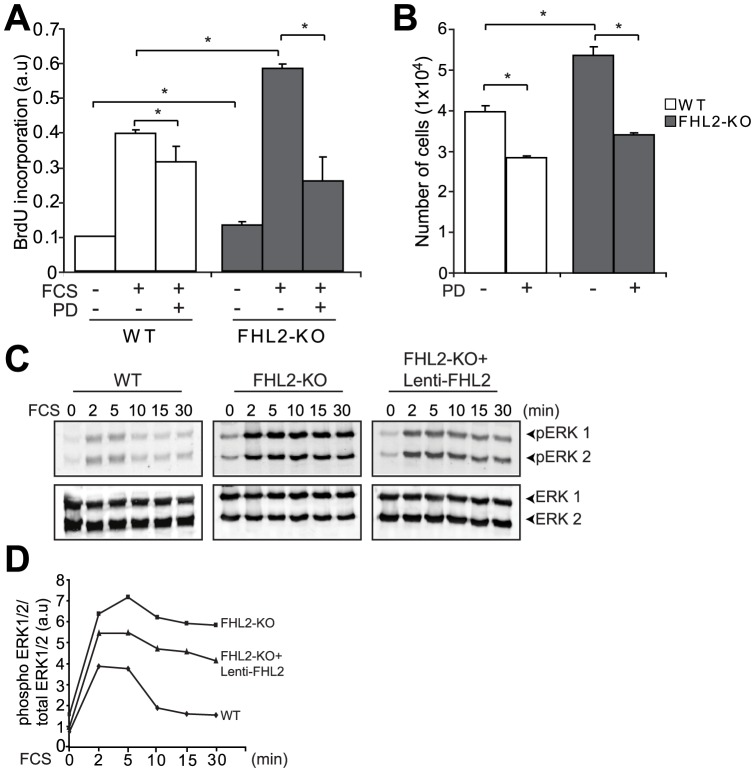
FHL2 deficiency enhances SMC proliferation via activation of ERK1/2. **A**, Serum-starved SMCs were stimulated with or without FCS and treated with or without PD98059 (ERK1/2 inhibitor, 25µM). Cells were pulse-labeled with BrdU to measure DNA synthesis. **B**, SMCs from WT and FHL2-KO were seeded at equal density. 1 day after seeding, cells were treated with or without PD98059 and cells were counted manually. **C–D**, Western blot analysis (C) and quantification (D) for pERK1/2 in serum-starved SMCs after overexpression with or with out FHL2 followed by FCS stimulation for the indicated time periods, showing enhanced and prolonged activation of ERK1/2 in FHL2-defeicient SMCs and reduced activation of ERK1/2 in FHL2-KO SMCs after overexpression of FHL2. Data represent means±SD. *P<0.05 for FHL2-KO versus WT.

Since we observed increased SMC proliferation in the lesions of FHL2-KO mice compared to lesions from WT mice, we investigated the role of FHL2 in proliferation of cultured aortic SMCs from WT and FHL2-KO mice. FHL2-KO SMCs exhibited increased cell proliferation in response to serum stimulation compared with WT SMCs, as demonstrated by higher BrdU incorporation (Fig. 3A) and increased numbers of cells (Fig. 3B). Previous studies showed that SMC proliferation and migration are controlled by MAPK signaling pathways and that FHL2 interacts with ERK2 [11], [13]. To demonstrate that FHL2 and ERK1/2 indeed interact in SMCs, we performed co-immunoprecipitation (co-IP) experiments. We found that FHL2 interacts with ERK1/2 in WT SMCs. As a control, we have also included FHL2-KO SMCs (Fig. S2). Next, we evaluated the activation of ERK1/2 in WT and FHL2-KO SMCs. FHL2-KO SMCs showed increased phosphorylation of ERK1/2 for a prolonged period of time up to 30 min, whereas ERK1/2 phosphorylation in WT SMCs was lower and more transient with optimal phosphorylation at 2–5 min (Fig. 3C–D). Consistent with this observation, pre-treatment of SMCs with PD98059, an ERK1/2 inhibitor, resulted in decreased proliferation of FHL2-KO SMCs compared with untreated cells (Fig. 3A–B). In addition, we show that overexpression of FHL2 using lentivirus decreases phosphorylation of ERK1/2 in FHL2 KO SMCs (Fig. 3C–D). The transduction efficiency of FHL2 lentivirus in WT and FHL2-KO SMCs was analyzed by qRT-PCR (Fig. S3). To further substantiate these *in vitro* findings, we also performed immunohistochemical stainings for phospho- ERK1/2 on the ligated vessels from WT and FHL2-KO. We show that phospho-ERK1/2 activation is increased in lesions from FHL2-KO compared to lesions from WT mice (Fig. S4). Taken together, these data indicate that the enhanced proliferation in FHL2-KO SMCs is regulated, at least partly, through enhanced activation of ERK1/2.

**Figure 4:**
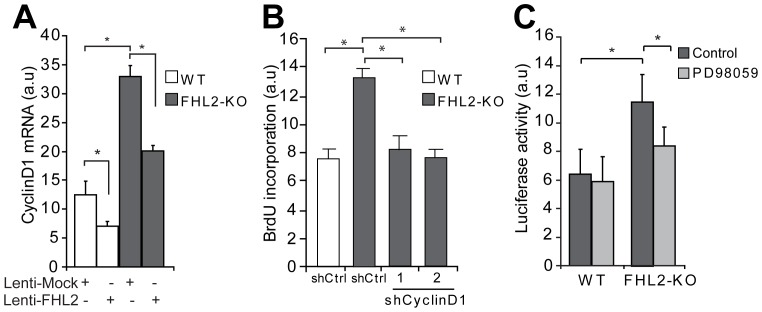
FHL2 regulates cell proliferation via modulation of CycinD1 expression. **A**, SMCs were transduced with lentiviral particles encoding FHL2 and assayed for CyclinD1 mRNA expression, showing that FHL2 inhibits its expression. **B**, Serum-starved WT SMCs were transduced with lentiviral particles encoding shCtrl, shCyclinD1 #1 and shCyclinD1 #2 and were pulse-labeled with BrdU to measure DNA synthesis. **C**, The CyclinD1 promoter-reporter plasmid showed higher induction in FHL2-KO SMCs stimulated with FCS than in WT SMCs. The ERK1/2 inhibitor PD98059 partly reduces this induction. Data represent means±SD. *P<0.05 for FHL2-KO versus WT.

Several lines of evidence implicated a role of FHL2 in the control of cell proliferation. Although there is no direct DNA binding capacity shown for FHL2, it has been demonstrated that FHL2 associates, most likely indirectly, with the CyclinD1 promoter and regulates its transcription in a cell-type dependent manner [23]. The expression of CyclinD1 mRNA was 2.5 fold higher in FHL2-KO SMCs than in WT SMCs (Fig. 4A). Furthermore, FHL2 overexpression in SMCs decreased CyclinD1 expression both in WT and FHL2-KO SMCs (Fig. 4A). To explore whether enhanced CyclinD1 expression is responsible for the increased proliferation observed in FHL2-KO SMCs, we performed BrdU incorporation assays after knock-down of CyclinD1. The knock-down efficiency of CyclinD1 as analyzed by qRT-PCR was 75–80% (Fig. S5A). Indeed, the enhanced cell proliferation of FHL2-KO SMCs was significantly reduced after knock-down of CyclinD1 by two different shRNA sequences (Fig. 4B). We also assessed CyclinD1 protein expression on the ligated vessels from WT and FHL2-KO, which show enhanced CyclinD1 expression in lesions from FHL2-KO compared to lesions from WT mice (Fig. S4). Consistent with these data, the luciferase activity of a CyclinD1 promoter luciferase-reporter was increased in FHL2-KO SMCs (Fig. 4C). Since the ERK1/2 pathway has been demonstrated to regulate CyclinD1 transcriptional activity in different cell types including SMCs, we investigated CyclinD1 promoter activity after pretreatment with PD98059. CyclinD1 promoter activity was reduced in FHL2-KO SMCs after pretreatment with PD98059. Collectively, these results imply that FHL2 affects SMC proliferation through enhanced activation of the ERK1/2-CyclinD1 signaling pathway.

### Enhanced migration of FHL2-deficient SMCs in vitro

**Figure 5:**
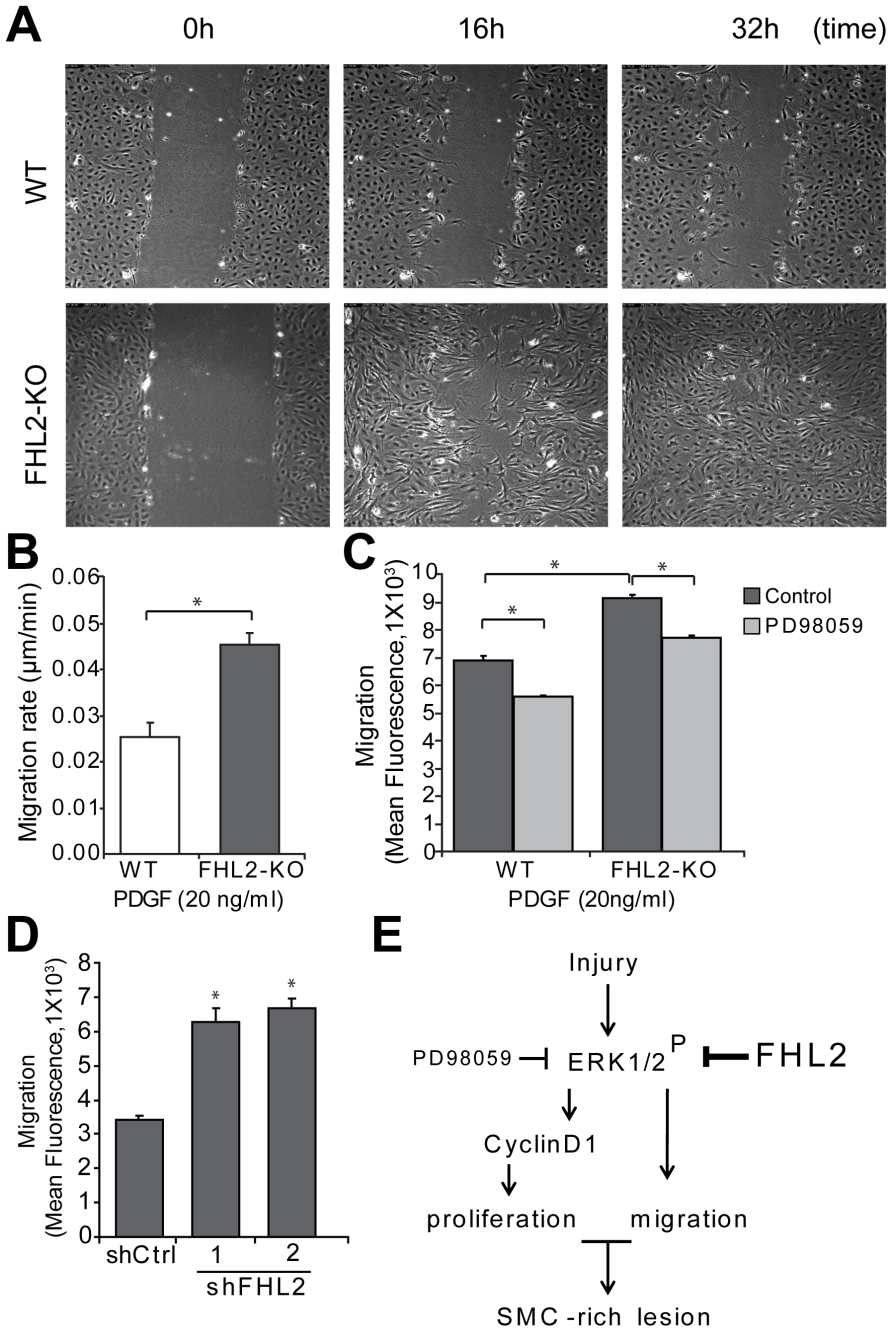
FHL2-KO SMCs migrate faster. **A**, A scratch was made in a confluent layer of serum-starved SMCs that were stimulated with PDGF (20 ng/ml). Images were captured every 10 min using a live cell microscope and representative images at 0, 16 and 32 h are shown. Movies of the movement are in the online supplement. **B**, Quantitative analysis of SMC migration in the scratch wound assay showing that FHL2-KO SMCs migrated 1.8 fold faster than WT SMCs. **C**, SMCs were treated with or without PD98059 and cell migration was evaluated using a trans-well assay. Cells were labeled with a fluorescent dye and seeded in the upper chamber. Cell migration was measured as fluorescence after 3 h. **D**, SMC migration was evaluated using a trans-well assay after knock-down of FHL2 using lentiviral particles encoding shCtrl, shFHL2#1 and shFHL2#2 in WT SMCs. Cell migration was measured as fluorescence after 3 h. Data represent means±SD. *P<0.05 for shCtrl versus shFHL2. **E**, Schematic representation of FHL2 function in the modulation of SMC-rich lesion formation. FHL2 modulates SMC-rich lesion formation by inhibiting proliferation and migration of SMCs via the ERK1/2-CyclinD1signaling pathway.

SMC migration is an important component of neointima formation and vascular remodeling [4], [8]. Results of previous studies in multiple cell culture systems have shown that FHL2 plays a vital role in cell migration [24], [25]. To explore the potential function of FHL2 in SMC migration, *in vitro* scratch wound assays were performed. Serum-starved WT and FHL2-KO SMCs were observed for migratory speed using time-lapse video microscopy. FHL2-KO SMCs migrated significantly faster into the scratch area in the given time period than WT SMCs, indicating that FHL2 deficiency increases the PDGF-induced migration rate of SMCs (Figs. 5A–B; see Videos S1–2). To further substantiate these results, we performed trans-well assays to measure SMC migration in serum-starved SMCs. In line with the scratch wound assays, FHL2-KO SMCs migrated significantly faster than WT SMCs (Fig. 5C). Since the ERK1/2 pathway has been shown to be important for cell migration, we tested SMC migration after pretreatment with PD98059. Indeed, migration of FHL2-KO SMCs was significantly reduced after treatment with PD98059 compared to control (Fig. 5C). To assess whether deletion of FHL2 in adult SMCs also affects cell migration, we performed knock-down experiments. The knock-down efficiency of FHL2 was analyzed by qRT-PCR (Fig. S5B). Knock-down of FHL2 in adult WT SMCs using shRNA also showed significant increase in SMC migration (Fig. 5D). Taken together, FHL2-deficient SMCs migrate faster than WT SMCs, involving activation of the ERK1/2 pathway. In Fig. 5E, we summarized our data in a schematic representation.

## Discussion

Restenosis is still one of the major limitations of angioplasty interventions in coronary arteries in spite of the development of drug-eluting stents. In-stent restenotic lesions are composed predominantly of proliferating SMCs with some infiltrated inflammatory cells [26], [27]. To study the underlying mechanism of this disease, we employed the mouse carotid artery ligation model, which involves endothelial cell activation, an inflammatory response in the vessel wall as well as migration and proliferation of SMCs [6], [18]. In the present study, we demonstrate that FHL2 deficiency exacerbates SMC-rich lesion formation through activation of these underlying processes in response to vascular injury. SMCs deficient for FHL2 showed a strong intrinsic induction of cellular proliferation and migration in culture. Furthermore, we observed enhanced activation of ERK1/2 and increased CyclinD1 expression in FHL2-KO SMCs, which promote SMC proliferation and migration.

Recently, it has been reported that deficiency of FHL2 may inhibit atherosclerosis after a cholesterol-enriched diet [17]. In that specific study vascular lesion formation was monitored in regular C57BL/6 mice, which develop very small atherosclerotic lesions that were visualized by electron microscopy and were shown to be composed predominantly of macrophages. The discrepancy with our study underscores the difference between these models of arterial disease, resulting in lesions composed of predominantly SMCs or inflammatory cells.

We and others have demonstrated that FHL2 is expressed in endothelial cells, but not in macrophages [11]. In endothelial cells, FHL2 has been shown to interact with sphingosine kinase-1, resulting in suppression of the VEGF signal transduction pathway and thus inhibition of angiogenesis [28]. In contrast, deficiency of FHL2 was also shown to impair angiogenesis in the aortic-ring culture assay [29]. In the current study we did not incorporate analyses to further delineate the contribution of FHL2-deficiency in endothelial cells during carotid artery ligation-induced lesion formation, which may require further studies.

The function of FHL2 in cellular proliferation and migration has so far been shown to be extremely cell-type dependent. Overexpression of FHL2 inhibits the growth of colon and liver cancer cell lines, whereas an opposite, growth-accelerating function has been associated with FHL2 based on experiments with fibroblasts of FHL2-KO mice [23], [30], [31]. Similarly, the migration of bone-marrow derived dendritic cells is reduced by FHL2, whereas the same factor promotes migration of skin fibroblasts [24], [25]. The ERK1/2 pathway has been implicated both in SMC migration and proliferation during neointima formation [32], [33], [34]. ERK activation is typically biphasic, with early ERK activation being associated with cell migration and later ERK activity being essential for cell proliferation [35]. FHL2 has been shown to physically interact with ERK2 and repress ERK1/2 activation in cardiomyocytes [13]. In line with those observations, we showed enhanced ERK1/2 activation in FHL2-KO SMCs, which may thus impact on both SMC growth and migration. In addition, FHL2 has been implicated in cell motility and contractility by interacting with proteins of focal adhesion structures, which we did not explore in SMCs and may require closer examination [9], [20].

Previously, we demonstrated that FHL2 interacts and inhibits the activity of the nuclear receptor Nur77 which was known to inhibit proliferation of SMCs [11]. To further delineate the function of FHL2 in Nur77 regulation, FHL2 was knocked down in SMCs overexpressing Nur77, which resulted in decreased proliferation in that specific study [11]. These data may seem in contrast with our current observations showing that FHL2-KO SMCs exhibit increased proliferation, however, FHL2-knockdown in WT SMCs does result in enhanced SMC proliferation similarly as in FHL2-KO SMCs (data not shown).

Labelette et al demonstrated that FHL2 stimulates CyclinD1 transcription in fibroblasts [23]. In contrast, Ng et al showed that FHL2 overexpression inhibits CyclinD1 expression in liver cancer cells [30]. In the current study, we found CyclinD1 expression significantly induced in FHL2-KO SMCs, which shows another example of the cell-type specific responses of FHL2. We further substantiated our observation by demonstrating that overexpression of FHL2 decreased CyclinD1 expression in SMCs. CyclinD1 transcriptional activity is modulated by the ERK1/2 pathway and we found indeed that the enhanced CyclinD1 transcriptional activity in FHL2-KO SMCs was reduced upon ERK1/2 inhibition. Our data support the conclusion that the enhanced SMC proliferation upon FHL2 deficiency coincides with stimulation of the cell cycle-regulating gene CyclinD1.

In summary, we demonstrate that FHL2 deficiency promotes the development of SMC-rich lesions after carotid artery ligation. The exacerbated lesion formation in FHL2-KO compared to WT mice involves enhanced proliferation and migration of SMCs deficient for FHL2 with higher expression of CyclinD1 through the ERK1/2 pathway (see Fig. 5E for the schematic representation). These findings indicate that FHL2 is an important mediator of SMC function with a beneficial function in vascular proliferative disease.

## Supporting Information

Figure S1 **Number of white-blood cells after carotid artery ligation.** White-blood cells were counted using coulter counter before (A) and after 4 weeks (B) of carotid artery ligation. Data are presented as mean±SD.
(PDF)

Figure S2 **FHL2 interacts with ERK1/2.** Whole cell extracts from WT and FHL2-KO SMCs were prepared and immunoprecipitated with anti-ERK1/2 antibody. Immunoprecipitated samples were resolved on 10% SDS-PAGE and analyzed by Western blotting with anti-FHL2 antibody. Data are representative of two independent experiments.
(PDF)

Figure S3 **FHL2 overexpression in SMCs.** SMCs were transduced with lentiviral particles encoding FHL2 and assayed for FHL2 mRNA expression.
(PDF)

Figure S4 **FHL2 deficiency enhances ERK1/2 activation and CyclinD1 expression in vivo.** Representative sections of ligated carotid arteries from WT and FHL2-KO mice were immunostained for phospho-ERK1/2 (top panels) and CyclinD1 (lower panels). M (media); I (intima).
(PDF)

Figure S5 **Knock-down efficiency of CyclinD1 and FHL2.** **A**, WT SMCs were transduced with lentiviral particles encoding shCtrl, shCyclinD1 #1 and shCyclinD1 #2. qRT-PCR was performed to assess knock-down of CyclinD1,showing about 70% knock-down of CyclinD1. **B**, WT SMCs were transduced with lentiviral particles encoding shCtrl, shFHL2#1 and shFHL2#2. qRT-PCR was performed to assess knock-down of FHL2, showing 55–60% knock-down of FHL2. Data represent means±SD. *P<0.05 for shctrl versus shCyclinD1 or shFHL2. The bar graphs represent results from at least 3 separate experiments.
(PDF)

Checklist S1 (DOC)

Videos S1 **FHL2-KO SMCs migrate faster in the scratch wound assay.** A scratch was made in a confluent layer of serum-starved SMCs that were stimulated with PDGF (20 ng/ml). Movies of WT were generated with a digital camera.
(MP4)

Videos S2 **FHL2-KO SMCs migrate faster in the scratch wound assay.** A scratch was made in a confluent layer of serum-starved SMCs that were stimulated with PDGF (20 ng/ml). Movies of FHL2-KO were generated with a digital camera.
(MP4)
