# Supplementary figures and images for: The LIM-Only Protein FHL2 Reduces Vascular Lesion Formation Involving Inhibition of Proliferation and Migration of Smooth Muscle Cells
Source: PLoS One. 2014 Apr 15;9(4):e94931. doi: 10.1371/journal.pone.0094931 (PMC3988136; doi:10.1371/journal.pone.0094931)

Figure S1

**A**

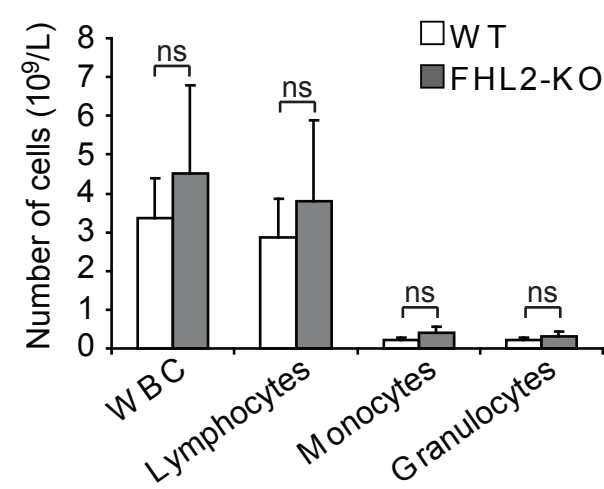

**B**

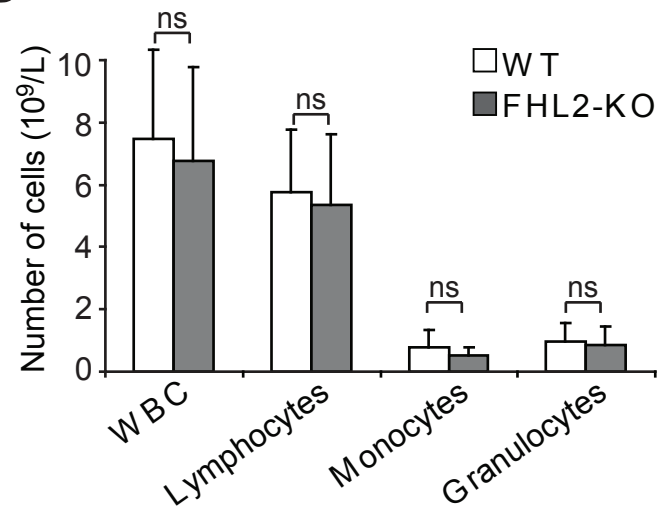

Supplement: Figure S1 — Number of white-blood cells after carotid artery ligation. White-blood cells were counted using coulter counter before (A) and after 4 weeks (B) of carotid artery ligation. Data are presented as mean±SD. (PDF) [file pone.0094931.s001.pdf]

**Figure S2**

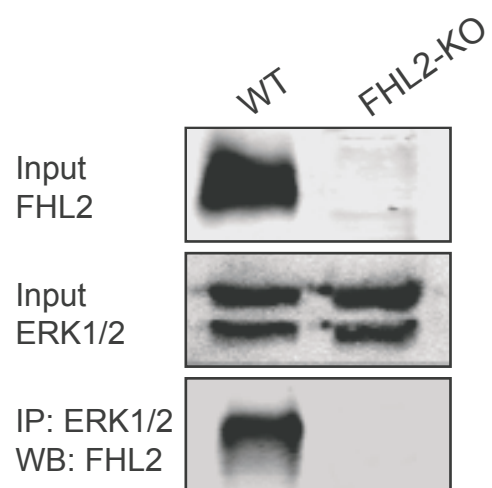

Supplement: Figure S2 — FHL2 interacts with ERK1/2. Whole cell extracts from WT and FHL2-KO SMCs were prepared and immunoprecipitated with anti-ERK1/2 antibody. Immunoprecipitated samples were resolved on 10% SDS-PAGE and analyzed by Western blotting with anti-FHL2 antibody. Data are representative of two independent experiments. (PDF) [file pone.0094931.s002.pdf]

**Figure S3**

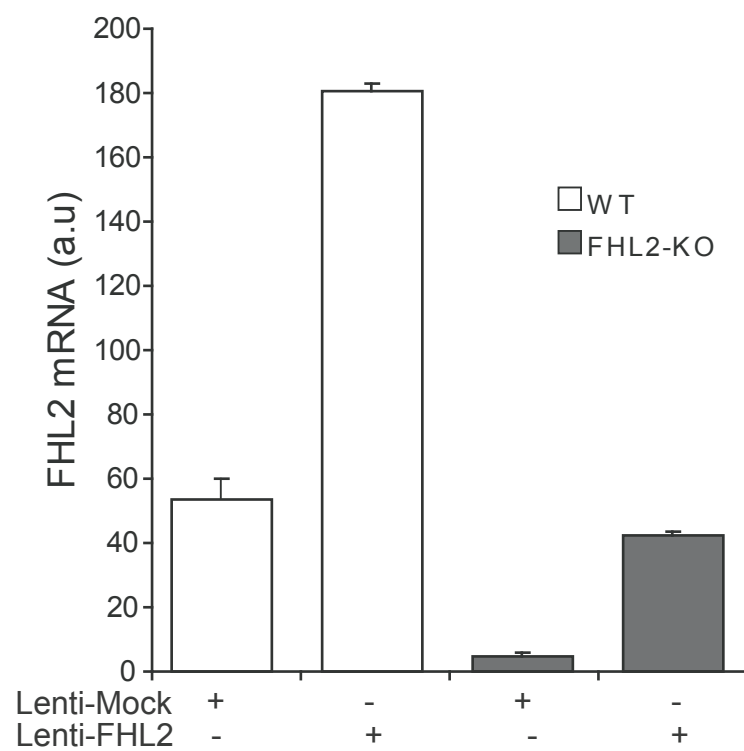

Supplement: Figure S3 — FHL2 overexpression in SMCs. SMCs were transduced with lentiviral particles encoding FHL2 and assayed for FHL2 mRNA expression. (PDF) [file pone.0094931.s003.pdf]

**Figure S4**

**A**

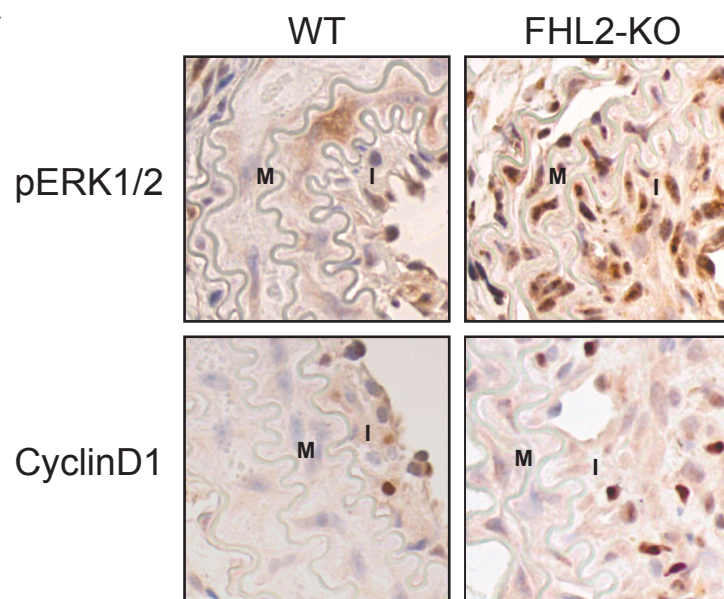

Supplement: Figure S4 — FHL2 deficiency enhances ERK1/2 activation and CyclinD1 expression in vivo. Representative sections of ligated carotid arteries from WT and FHL2-KO mice were immunostained for phospho-ERK1/2 (top panels) and CyclinD1 (lower panels). M (media); I (intima). (PDF) [file pone.0094931.s004.pdf]

**Figure S5**

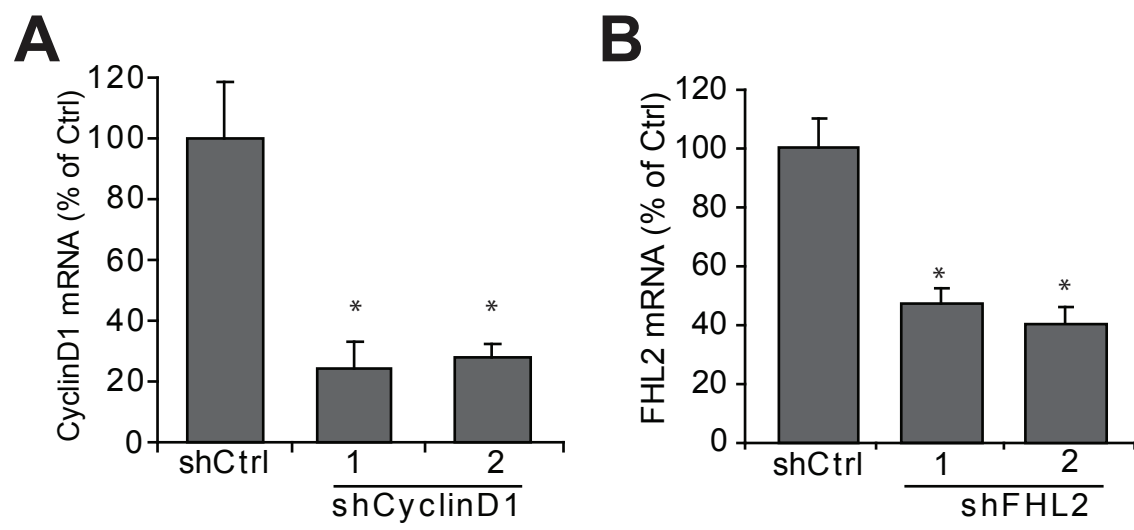

Supplement: Figure S5 — Knock-down efficiency of CyclinD1 and FHL2. A, WT SMCs were transduced with lentiviral particles encoding shCtrl, shCyclinD1 #1 and shCyclinD1 #2. qRT-PCR was performed to assess knock-down of CyclinD1,showing about 70% knock-down of CyclinD1. B, WT SMCs were transduced with lentiviral particles encoding shCtrl, shFHL2#1 and shFHL2#2. qRT-PCR was performed to assess knock-down of FHL2, showing 55–60% knock-down of FHL2. Data represent means±SD. *P<0.05 for shctrl versus shCyclinD1 or shFHL2. The bar graphs represent results from at least 3 separate experiments. (PDF) [file pone.0094931.s005.pdf]
